# Application of Cobas 6800 HPV testing and qPCR utilizing three genomically-optimized primer sets for detecting HPV DNA in the blood of HPV Positive dysplasia and cancer patients

**DOI:** 10.1371/journal.pone.0354671

**Published:** 2026-08-25

**Authors:** Heba H. Mostafa, Amary Fall, Stéphanie Gaillard, Carole Fakhry, Zubair Khan, Tanguy Seiwert, Hao Wang, Hua-Ling Tsai, Amanda N. Fader, Charles A. Leath, Warner K. Huh, Rebecca C. Arend, Ya-chea Tsai, Joseph A. Califano, Deyin Xing, T.-C. Wu, Richard B. S. Roden

**Affiliations:** 1 Department of Pathology, Johns Hopkins School of Medicine, Baltimore, Maryland, United States of America; 2 Department of Oncology, Johns Hopkins School of Medicine, Baltimore, Maryland, United States of America; 3 Department of Gynecology/Obstetrics, Johns Hopkins School of Medicine, Baltimore, Maryland, United States of America; 4 Department of Department of Otolaryngology Head and Neck Surgery, Johns Hopkins School of Medicine, Baltimore, Maryland, United States of America; 5 Department of Biostatistics, Johns Hopkins School of Medicine, Baltimore, Maryland, United States of America; 6 Division of Gynecologic Oncology, University of Alabama at Birmingham, Birmingham, Alabama, United States of America; 7 Moores Cancer Center, University of California San Diego, San Diego, California, United States of America; Istituto Nazionale Tumori IRCCS Fondazione Pascale, ITALY

## Abstract

Use of quantitative polymerase chain reaction (qPCR) to measure viral DNA in plasma has promise for the detection and/or monitoring of high-risk human papillomavirus (hrHPV)-associated cancers. We examined Roche’s cobas 6800 HPV test for detection of hrHPV in plasma or peripheral blood mononuclear cells (PBMC) of patients with HPV-associated dysplasia or cancer. In our pilot study, this assay detected hrHPV DNA in 1 mL pre-treatment plasma of patients with advanced (stage II-IV) cervical cancer, but not stage I or dysplasia cases, or in PBMC. Plasma of oropharyngeal cancer patients also tested positive in most cases. Seeking to enhance sensitivity, we re-tested these pilot samples with qPCR employing three published primer sets (E7, BR E1-5, HR E5L2-4) selected for improved detection of HPV16 in cancer. This triplex assay detected quantifiable levels of HPV16 in pre-treatment plasma of advanced (stage II-IV) cervical cancer cases, and lower levels in some stage I and dysplasia cases. In serial sampling of HPV16 + cervical cancer patients post-treatment with no evidence of disease over ~2 years, no reproducible signal was detected by cobas HPV testing, whereas the triplex qPCR sporadically detected HPV DNA in plasma and PBMC samples of a subset of these patients.

## Introduction

HPV infections are epithelial rather than systemic and are typically very localized [1]. Approximately a dozen high-risk HPV (hrHPV) genotypes are a necessary causes of about 5% of all cancers worldwide, primarily cervical cancer [2]. HPV also causes other anogenital cancers (vaginal, vulvar, anal and penile) and a subset of oropharyngeal cancers. Among hrHPV genotypes, HPV16 accounts for 50–60% of cervical cancers, with HPV18 contributing an additional 20%. In contrast, HPV16 is detected in 80–90% of the other anogenital and oropharyngeal cancer tissues.

The Papanicolaou (Pap) test allows for the detection and treatment of pre-cancerous cervical intraepithelial neoplasia (CIN2/3). The implementation of national cervical screening programs can reduce the burden of cervical cancer by 80% [3], but has been mostly limited to high income countries. Co-testing for hrHPV nucleic acid can improve the sensitivity for detecting dysplasia and a negative test has higher negative predictive value as compared to cytology. Indeed, there is a movement toward primary cervical cancer screening by HPV testing [4,5].

HPV vaccines are highly effective in preventing infection, but have no impact once HPV is established [6]. Cervical dysplasia and even localized invasive cervical cancer can generally be cured by surgical intervention, such as cold knife conization or hysterectomy respectively [7]. Surgery is also used to treat dysplasia at other anogenital sites and for locally advanced oropharyngeal cancers. Most advanced cancers require chemoradiotherapy and biologic therapy with monoclonal antibodies targeting VEGF and/or immune checkpoints (PD-1).

Currently, there are no licensed blood biomarkers to inform cancer treatment decisions or screening for HPV+ cancer. Since metastatic cancer cells frequently shed their genomic DNA into the bloodstream, there is potential to use plasma detection of circulating free hrHPV DNA as a blood biomarker for screening, prognosis and/or monitoring of treatment. Early studies deployed qPCR with a titrated standard to provide quantitation. More recently, digital PCR (dPCR) and next generation sequencing (NGS) have been used to improve sensitivity for detection [8–10], including in several commercial assays [11–13]. These newer technologies bring increased technical complexity and cost, but their benefit to clinical outcomes and utility for screening remain unproven.

The qPCR diagnostics have been implemented globally, including as robust robotic systems at high throughput and relatively low cost, for detection of infections. Here we examine the potential of one such automated system, Roche cobas 6800, that is widely implemented for use with cervical cytology specimens, but here utilized with modified protocol (non-validated) to detect HPV DNA in human plasma to screen for and/or monitor HPV+ cancer burden [14]. This is compared to a qPCR assay, previously developed by Saito et al, that combines three primer sets rationally-designed to detect HPV16 + cancers based on read density of whole genome sequencing of HPV16 + head and neck squamous cells cancers (HNSCC) and the detection of HPV DNA in a high proportion of tumors [15]; hereafter it is referred to the “Triplex” assay.

## Materials and methods

### Human blood and tissue specimens

The protocol was approved by the Johns Hopkins University Institutional Review Board (NA_00017599). This study was conducted in compliance with the US Common Rule. All participants provided written informed consent. Samples were anonymized prior to data collection. Patients ≥18 years old undergoing surgery in the Division of Gynecologic Oncology or Head and Neck Surgery at the Johns Hopkins Hospital. Additional samples were evaluated from patients enrolled on a phase I clinical trial (NCT02405221) of an HPV16 therapeutic vaccine at Johns Hopkins University or University of Alabama at Birmingham [16]. Briefly, patients were enrolled with the following criteria: females, age ≥ 18 years with a diagnosis of stage IB1-IVA cervical cancer, who had completed definitive treatment within the past 12 months, and had no evidence of disease recurrence based on imaging and clinical assessments within 8 weeks of enrollment. Diagnostic tumor specimens were required to be positive for HPV16 by RNAScope in situ hybridization. The study recruitment period was 04/04/2019–31/01/2025.

Blood was collected from patients in 10mL EDTA-treated tubes by standard venipuncture, and plasma separated by centrifugation for 10 min at 600 x *g* from the cellular components*.* Plasma aliquots of 1 mL were stored at −80°C until use. The remaining cell pellet was resuspended in ACK buffer and after lysis of the red blood cells, white blood cells (WBC) were collected by centrifugation, washed in PBS, and stored in 2 aliquots at −80°C. Separately, PBMC were purified from heparinized blood by Ficoll separation and frozen viably in 90% FCS/10% DMSO. Plasma of normal human volunteers was purchased from Lampire, Pipersville, PA. An oral rinse with 10mL normal saline was collected from a patient with HPV+ oropharyngeal cancer and centrifuged at 2500 rpm for 10 min. The pellet was resuspended in 1 mL saline, and 0.5 mL aliquots snap frozen and stored at −80°C [13].

Formalin-fixed paraffin-embedded tumor and corresponding normal tissues were evaluated by hematoxylin and eosin staining and subsequently were macrodissected (with tumor elements accounting for about 60% or more of the section area). Genomic DNA was extracted using a QIAamp DNA formalin-fixed paraffin-embedded Tissue Kit with an adopted protocol (Qiagen). Slides bearing paraffin-embedded tissue were first baked at 68°C for 20–30 seconds; the tissue was deparaffinized 3 times with xylene, and residual xylene was removed by washing through serial dilutions of ethanol. The rest of the procedure followed the manufacturer’s instructions.

### qPCR assays

Banked plasma and Ficoll-purified PBMC samples were tested in the cobas 6800 system (Roche) in a CLIA environment. Alternatively, DNA was purified from banked plasma and WBC and Ficoll-purified PBMC samples were tested in the Triplex qPCR assay using E7, BR E1-5, and HR E5L2-4 primers in a CLIA environment [15]. To assess sensitivity, plasma samples from healthy donors were doped with known amounts of purified genomic plasmid clones of individual HPV genotypes. These plasmid clones were provided by the International Human Papillomavirus Reference Center (https://www.hpvcenter.se/), expanded in E. coli DH5α, purified using Endo-free maxiprep kits (Qiagen) and sequence verified.

### Statistical analyses

Study measurements were summarized primarily using descriptive statistics. Concordance between assays was reported, and differences in positivity rates between paired assays were assessed using McNemar’s test. Temporal changes in the highest intensity measured from plasma and WBC samples based on Saito primer sets was also assessed. Differences in the changes from baseline, as well as associations between changes from baseline to follow-up, and the corresponding clinical disease characteristics (such as stage and histologic type), were assessed using the Wilcoxon test, as appropriate for one-sample or two-sample comparisons.

## Results

Initial studies centered on determining the efficiency and sensitivity to detect HPV genomic DNA in plasma. A 10-fold dilution series of recombinant HPV16 genomic plasmid DNA (10E6.7-10E3.7 copies) was spiked to 1 mL aliquots of plasma. Contrived samples were tested in triplicate with the cobas 6800 HPV assay which detects 14 hrHPV types including HPV16, HPV18, or other hrHPV, and includes an internal control human genomic sequence (human β-globin gene) [14]. The C_t_ value was obtained for each reaction and plotted against the log10 number of plasmid copies/mL. Based upon the slope of −3.423 (95% CI −3.53 to −3.316) the PCR reaction was 96% (95% CI 92% to 100%) efficient suggesting no interference with the PCR reaction by residual components of the plasma. The limit of detection was 100 copies/ mL for HPV16 (S1A Table) and 100 copies/ mL for HPV18 (S1B Table), consistent with prior reports [14].

Additionally, a verification panel comprising inactivated pellets of cells containing HPV16, HPV18, HPV31, HPV45, and HPV68 were spiked into HPV-negative plasma samples and tested at 10-fold dilutions (7–12 replicates) with the cobas 6800 system (S2 Table). The observed analytical sensitivity for cell-derived HPV16 and 18 was 10 copies/mL, and 100 copies/mL for other hrHPV. Next, detection of cell-free HPV DNA in plasma of patients with HPV-associated lesions using the cobas 6800 system was examined (Table 1). One mL aliquots of banked plasma collected from 34 female patients about to undergo surgery for anogenital pre-cancer and cancer was tested. When available, DNA extracted from a single slide from the FFPE tissue derived from lesion removed during their surgery was also tested. A positive result was obtained for 14 of the 27 tissue samples, including 9 HPV16 + , 1 HPV18+ and 4 other hrHPV + . Five of 25 plasma samples were invalid due to failure to amplify the human genomic DNA. HPV16 was detected in two of the remaining 20 plasma samples. The genotype detected in plasma matched the genotype detected in the histopathologic samples. Both positives were of patients with cervical squamous cell carcinoma, one was International Federation of Gynecology and Obstetrics (FIGO) stage IIB and the other was stage IV, the only two advanced cases tested. Notably, by cobas 6800 testing of the plasma, none of 8 stage I cervical cancers were positive for HPV DNA. Plasma derived from patients with HPV+ intraepithelial neoplasia through adenocarcinoma in situ also tested negative for HPV by cobas. The positive plasma samples were restricted to patients whose lesion was HPV16 + , and there were no false positives.

**Table 1:** Detection of HPV DNA by cobas 6800 or Triplex qPCR in blood and FFPE tissue specimens obtained from patients prior to surgery.

Pathology data			Tissue/cobas			Tissue/Saito			Plasma/cobas			PBMC/cobas			Plasma/Saito		
Diagnosis	Stage	ISH	Hr	16	18	E7	BRE1–5	HRE5L2-4	Hr	16	18	Hr	16	18	E7	BRE1–5	HRE5L2-4
VaIN2–3	precursor		neg	**36.24**	neg	**1387**	**3611**	**6981**	neg	neg	neg	neg	neg	neg	neg	neg	neg
VIN3	precursor		neg	neg	neg	**903**	**2499**	**7466**	Inv	Inv	Inv	neg	neg	neg	4.9*	neg	1.6*
VIN3	precursor		**32.06**	neg	neg	neg	neg	neg	neg	neg	neg	neg	neg	neg	neg	neg	neg
VaIN3/CIN2	precursor		neg	**31.26**	neg	**24090**	neg	**68.8**	neg	neg	neg	neg	neg	neg	6.0*	neg	neg
cervical HSIL	precursor		**30.62**	neg	neg	neg	neg	1.5*	neg	neg	neg	neg	neg	neg	neg	neg	13*
HSIL/CIN3	precursor		neg	neg	neg	neg	neg	neg	neg	neg	neg	neg	neg	neg	neg	neg	neg
HSIL/CIN3	precursor		neg	neg	neg	neg	neg	neg	neg	neg	neg	neg	neg	neg	neg	neg	neg
CIN3	precursor		neg	neg	neg	0.29*	neg	neg	Inv	Inv	Inv	neg	neg	neg	3.3*	neg	neg
cervical HSIL	precursor					neg	neg	neg	Inv	Inv	Inv	neg	neg	neg	neg	neg	neg
CIN3	precursor		neg	**37.61**	neg	0.24*	neg	4.8*	neg	neg	neg	neg	neg	neg	neg	neg	neg
cervical AIS	in situ		neg	**38.42**	neg	0.13*	neg	neg				neg	neg	neg	neg	neg	neg
cervical AIS	in situ		neg	neg	**36.76**	6.4*	neg	0.87*	neg	neg	neg	neg	neg	neg	neg	neg	neg
cervical AIS	in situ		**33.96**	neg	neg	neg	neg	neg	Inv	Inv	Inv	neg	neg	neg	neg	neg	neg
vulvar SCC	unknown		neg	neg	neg	neg	neg	neg	neg	neg	neg	neg	neg	neg	neg	neg	neg
vulvar SCC	unknown	hrHPV-ve	neg	neg	neg	neg	neg	neg	neg	neg	neg	neg	neg	neg	neg	neg	neg
vulvar SCC	unknown		neg	neg	neg	neg	2*	neg	neg	neg	neg	neg	neg	neg	neg	neg	neg
vulvar SCC	unknown		neg	neg	neg	5.7*	15*	neg	Inv	Inv	Inv	neg	neg	neg	neg	10*	15*
vulvar SCC	unknown		neg	neg	neg	**1975**	2.1*	neg	neg	neg	neg				neg	neg	neg
vulvar SCC	unknown	HPV16-ve	neg	neg	neg	neg	neg	neg	neg	neg	neg	neg	neg	neg	neg	neg	neg
vulvar adenoid cystic carcinoma	unknown		neg	neg	neg	neg	neg	neg	neg	neg	neg	neg	neg	neg	neg	neg	neg
recurrent vulvar SCC	unknown								neg	neg	neg	neg	neg	neg	neg	neg	neg
vulvar SCC	stage IB					neg	neg	neg	neg	neg	neg	neg	neg	neg	48*	neg	neg
vaginal ADC	stage II		neg	neg	neg	0.59*	4.3*	neg	neg	neg	neg	neg	neg	neg	neg	neg	neg
cervical SCC	stage IA1 pT1a1N0MX					neg	neg	neg	neg	neg	neg	neg	neg	neg	neg	neg	neg
cervical SCC	stage IA2 pT1a2N0MX								neg	neg	neg	neg	neg	neg	neg	neg	neg
endocervical ADC	stage 1B1 pT1b1N0	HPV16+	neg	**35.61**	neg	**946**	neg	11.6*	neg	neg	neg	neg	neg	neg	neg	neg	neg
cervical SCC	stage IB pT1bN0MX	HPV16+				**7546**	**80381**	**204385**	neg	neg	neg	neg	neg	neg	neg	13*	13*
cervical SCC	stage IB1 pT1b1N0MX		neg	**36.54**	neg				neg	neg	neg	neg	neg	neg	6.0*	neg	6.0*
cervical SCC	stage IB1 pT1b1N0MX		**28.19**	neg	neg	neg	neg	neg	neg	neg	neg	neg	neg	neg	neg	neg	neg
cervical mucinous ADC	stage 1B1 pT1b1N1MX	hrHPV-ve	neg	neg	neg	neg	neg	neg	neg	neg	neg	neg	neg	neg	31*	neg	neg
cervical SCC	stage IB2 pT1b2N1MX					neg	1.9*	neg	neg	neg	neg	neg	neg	neg	neg	neg	neg
cervical SCC	stage 2B pT2bN0MX		neg	**30.93**	neg	5.6*	**1093**	neg	neg	**34.21**	neg	neg	neg	neg	**380**	**36000**	neg
cervical SCC	stage IV	HPV16+	neg	**37.27**	neg				neg	**37.35**	neg	neg	neg	neg	71*	**430**	**610**
metastatic ADC	stage IV	hrHPV-ve	neg	**36.97**	neg	60*	**827**	neg	neg	neg	neg	neg	neg	neg	neg	neg	neg

The pathology diagnosis, stage information and results of testing for HPV16 or hrHPV by in situ hybridization (ISH) abstracted from the medical record is shown in the first three columns respectively. Testing of plasma (1mL) or PBMC (0.5-1x10^7^) or FFPE tissue derived from a single slide by cobas 6800 for hrHPV (Hr), or HPV16 (16) or HPV18 (18) is presented as the Ct value, negative (neg), or invalid (inv) if the internal control failed, or blank if not done. For detection by the three primers sets (E7, BRE1-5 and RE5L2-4), data are presented as copies/mL. *Copies/mL of <100 are below the lower limit of quantification (100).

Saito *et al*. used genomic data from CaSki cells and oropharyngeal cancer sequence databases to select three primer/probe sets targeting HPV16 between the E5 and L2 regions (HR E5L2-4) that exhibited the highest read density with NGS, a universally present E1 region (BR E1-5), and E7 [15]. Our prior assay development determined an analytical measurement range between 100 and 10^7^ copies.

We utilized these three primer/probe sets for testing the same plasma and FFPE samples (**Table 1**). Five FFPE tissues that tested negative with the cobas HPV assay, were positive by at least one of the Triplex qPCR primer sets [15]. While this suggests additional sensitivity, there were other tissue samples (n = 3) that were positive by the cobas assay, but negative by all three Triplex qPCR primer sets. These differences may reflect targeting of all hrHPV by the cobas assay, while the Triplex qPCR primer sets were designed to target HPV16 [15]. In addition, each assay amplifies a different region of the HPV genome. All 7 tissues positive by cobas, were also positive by at least one of the Saito *et al*. primer sets. Only 1 of the 4 tissues positive by cobas for other hrHPV, was also positive by at least one of the Triplex qPCR primer sets. Two CIN3 samples were negative by both assays, which might reflect integration by a hrHPV type other than HPV16 wherein the L1 gene is lost or disrupted, or sampling/sample quality issues.

Analysis of the plasma by all three Triplex qPCR primer sets [15] revealed quantifiable levels of HPV in a patient with stage IIB cervical cancer and a second with stage IV cervical cancer (tissues and plasma were positive by the cobas test). This suggests that the combination of the Triplex qPCR primer sets is at least as sensitive as the cobas HPV test for HPV16. Indeed, the Triplex qPCR primer sets also were positive, but below the lower limit of quantification [15], in plasma of several patients with stage I cervical cancer and vulvar SCC that were negative by the cobas HPV test. Furthermore, patients with positive plasma had corresponding positive lesion tissue samples, providing support that the low positive plasma detections are true positives.

**Fig 1:**
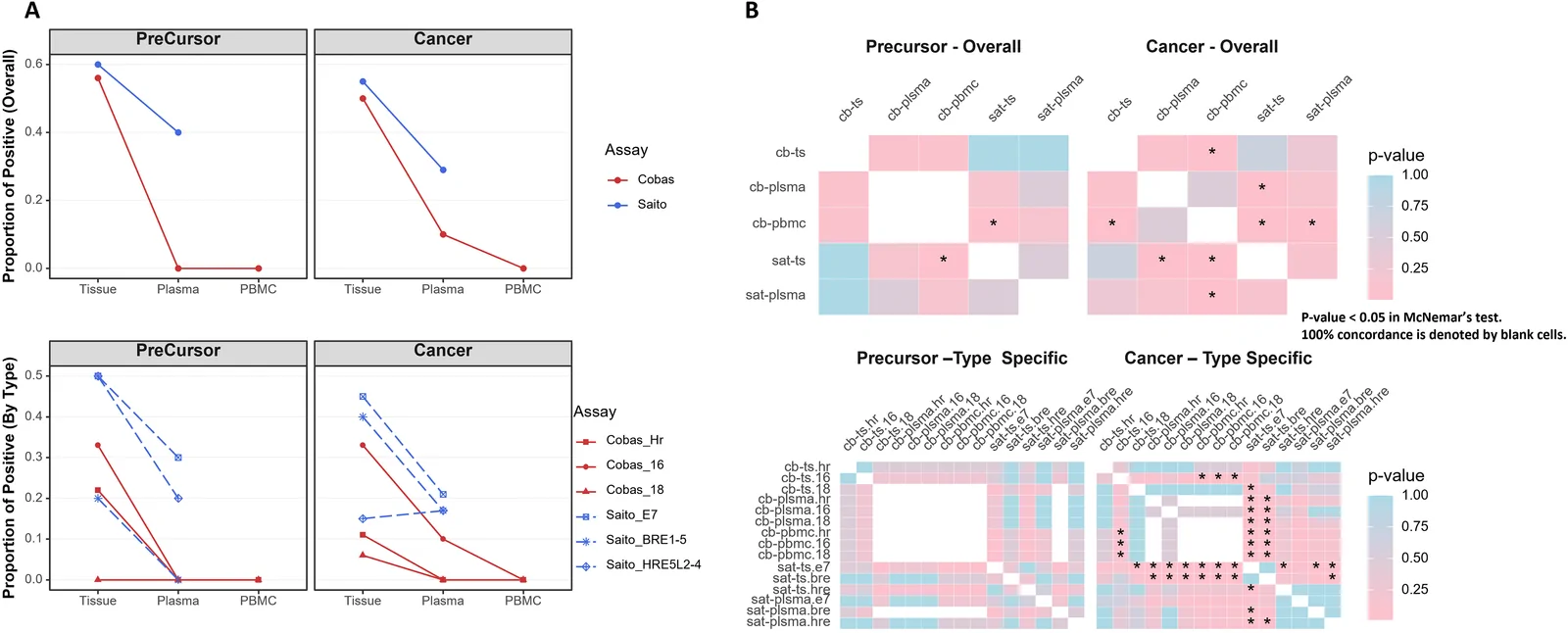
Comparison of the proportion of tissue, plasma and PBMC samples detected obtained at diagnosis from patients with anogenital cancers and their precursors. Data from Table 1 was analyzed. A) Overall, the Triplex qPCR assay (blue) detected more positivity than the Cobas 6800 HPV test (red). B) McNemar’s test showed a significant difference in positivity rates in tissue between qPCR using the E7 primer set versus the Cobas HPV test (vs. tissue 18, Cobas plasma all types, and Cobas pbmc all types) in paired samples, indicating that the assays did not classify samples similarly with respect to positive versus negative results.

Overall, the Triplex qPCR assay detected more positivity than the Cobas 6800 HPV test, with the E7 and BRE1–5 primers showing greater sensitivity than the HPE5L2-4 primers (Fig 1A). Application of McNemar’s test revealed a significant difference in positivity rates in tissue between qPCR using the E7 primer set versus the Cobas HPV test (vs. tissue 18, Cobas plasma all types, and Cobas PBMC all types) in paired samples, indicating that the assays did not classify samples similarly with respect to positive versus negative results. This likely reflects the presence of E7 in all HPV infected cells, whereas the L1 gene (targeted by the Cobas HPV assay) is often deleted in cervical cancers, and that the Triplex primers are designed to detect HPV16 whereas the Cobas HPV test targets all hrHPV types.

Therefore, we compared the cobas 6800 HPV test with the Triplex qPCR [15] for detection of HPV DNA in the plasma of 14 HPV16 + cervical cancer patients (Table 2 and S3 Table). These 14 patients were within 12 months of completing standard of care treatment and they exhibited no clinical evidence of disease when enrolled in a phase I vaccination clinical trial (NCT02405221) [16]. In the two-year trial, none of the patients showed clinical evidence of recurrence, although 2/14 patients (Table 2) were lost to follow up (after months 6 and 12 respectively). During this 2-year period, up to 7 blood samples were collected. Only one of the 82 plasma samples tested was invalid with cobas (1.2%). This is a much lower rate compared to the 20% for the other sample sets implying that how the sample is collected or processed may significantly impact the invalid rate. Notably, there were no visible signs of hemolysis in the trial samples, and their average long term storage was less than the earlier plasma sample sets (0–2 years versus 13–19 years in sample set of Table 1, with some evidence of hemolysis).

**Table 2:** Demographics of HPV16 + cervical cancer patients enrolled in phase I study and level of HPV DNA detected in serial blood samples.

Subject	Race	Ethnicity	Primary	Histology	Grade	Stage	Treatment	Weeks of follow-up	BL	M1	M2	M3	M6	Y1	Y2
1	Black	Non-Hispanic	Endocervix	Small cell carcinoma and adenosquamous carcinoma	No Comment	IB1	Surgery and adjuvant chemoradiation	104	0	0	0	83	**164**	**209**	48
2	Other	Hispanic	Endocervix	Adenocarcinoma	No Comment	IB1	Surgery	104	**146**	0	0	0	0	0	0
3	White	Non-Hispanic	Endocervix	Adenocarcinoma	Moderately differentiated	IB1	Surgery	104	**754**	96	45	**2573**	95	0	0
4	Black	Non-Hispanic	Endocervix	Squamous cell carcinoma	Moderately differentiated	IIA1	Chemoradiation	104	45	0	**125**	32	0	21	
5	Black	Non-Hispanic	Cervix, NOS	Squamous Cell Carcinoma	Moderately differentiated	IB2	Chemoradiation	104	0	0	63		0	75	0
6	Asian	Non-Hispanic	Cervix, NOS	Squamous cell carcinoma	Unknown	IA2	Surgery and adjuvant radiation therapy	104	0	0	0	0	0	0	0
7	White	Non-Hispanic	Cervix, NOS	Squamous cell carcinoma	Moderately differentiated	IIB	Chemoradiation	26	0	0	0	0	0		
8	White	Non-Hispanic	Cervix, NOS	Squamous cell carcinoma	Unknown	IB1	Chemoradiation	104	0	0	12	0	0	0	0
9	White	Non-Hispanic	Cervix, NOS	Adenocarcinoma	Well differentiated	IB1	Surgery	104	34	0	0	0			
10	White	Non-Hispanic	Endocervix	Squamous cell carcinoma	Moderately differentiated	IB2	Chemoradiation	104	**342**	0	0	0	0	0	0
11	White	Non-Hispanic	Endocervix	Adenocarcinoma	Well differentiated	IIA2	Chemoradiation	104	0	0	0	0	43		
12	Black	Non-Hispanic	Endocervix	Adenocarcinoma	Well differentiated	IB1	Surgery	104	0	0	0	**19,854**			
13	White	Non-Hispanic	Endocervix	Adenocarcinoma	Unknown	IIIC1	Surgery and adjuvant radiation therapy	104		0	0	0			
14	White	Non-Hispanic	Cervix, NOS	Squamous cell carcinoma	Poorly differentiated	IB1	Surgery	104	0	0	0	80			

Fourteen HPV16 + cervical cancer patients, who had no clinical evidence of disease within 12 months of completed standard of care treatment, were enrolled in a phase I clinical trial (NCT02405221) testing vaccination with TA-CIN, a fusion protein comprising HPV16 L2E7E6 [16]. Blood was collected at baseline (BL) pre-vaccination, and at Months 1, 2, 3 and 6 and Years 1 and 2 post-vaccination (M1, M2, M2, M6, Y1 and Y2 respectively). Patients were followed for clinical recurrence for up to 108 weeks. Samples of plasma (1mL) or WBC isolated from 5mL blood were tested with either the cobas 6800 HPV test or the Triplex qPCR primer sets (E7, BR E1-5 and HRE5L2-4). The highest number of copies of HPV16 DNA detected in any sample is presented in bold if above the lower limit of quantification described by Saito et al. Full details of the individual testing for each sample type and primer set is provided in S3 Table.

**Fig 2:**
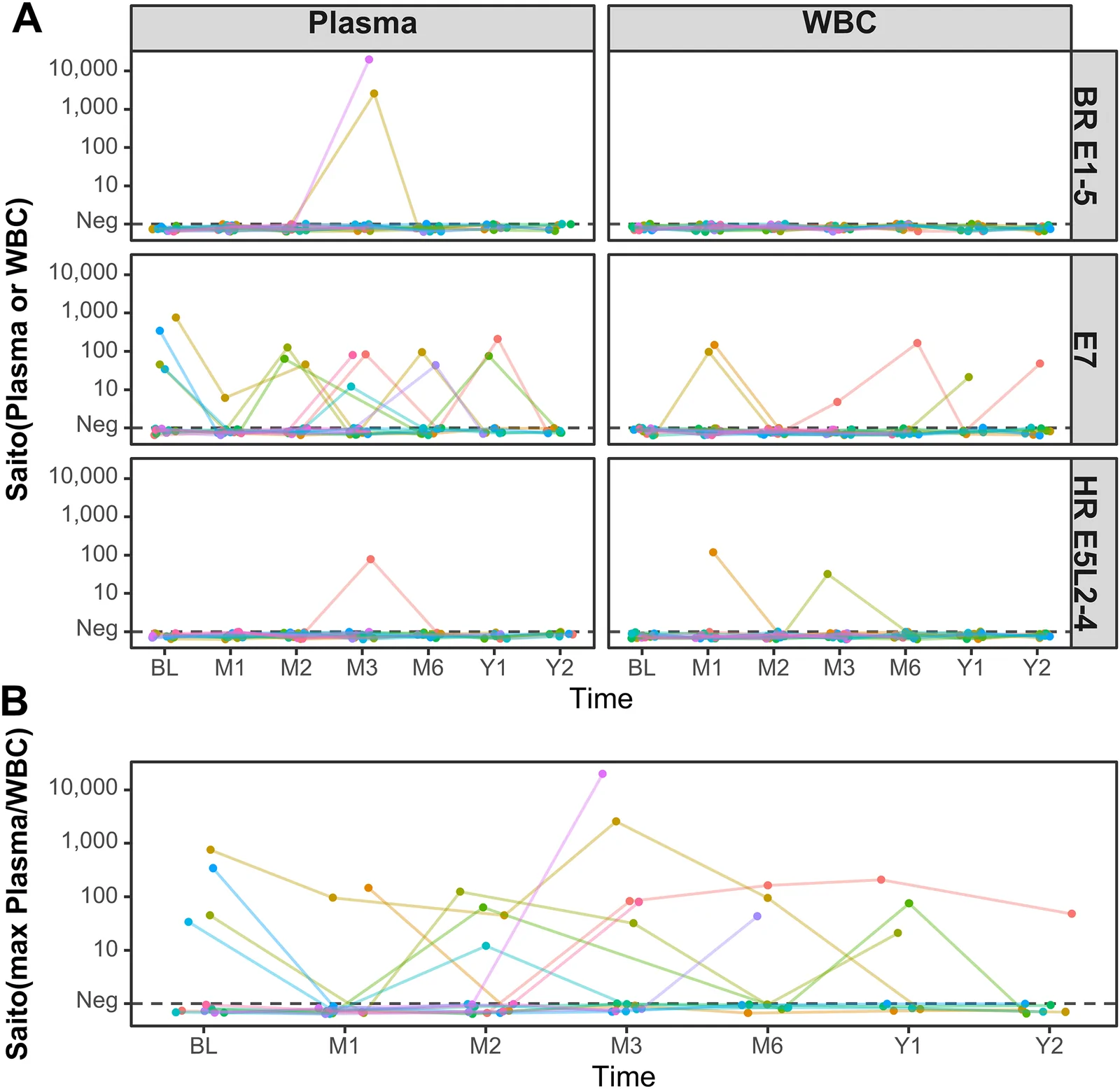
Scatter plot of longitudinal signal in blood samples of 14 cervical cancer patients using the Triplex qPCR assay. A) values in plasma and in WBC samples; B) maximum HPV DNA copy number values detected in either plasma or WBC samples using Saito et al. primer sets are presented for each time point.

Testing of the WBC samples from the same study timepoints with the Triplex qPCR revealed a lower percentage of positives, range from 0–12% over time across all primers, compared to testing the plasma samples, which ranged from 0–29% (Fig 2A and detailed in S3 Table). The concordance of results between plasma and WBC samples for at least one Triplex qPCR. primer sets ranged from 63% to 100% over time (S4 Table, Fig 2A). Regardless of primer sets or sample type, 46% of patients tested positive at baseline, whereas only 7% of patients tested positive after one month. Among positive patients at baseline, 83% (95% CI: 44–99%) of patients converted to negative status after one month (Fig 2). The highest copy number at month 1 was significantly different than the baseline (p = 0.04); however, other time points did not demonstrate a significant difference when compared to the baseline (S5 Table).

The highest HPV DNA copy number detected in plasma or WBC (S3 Table), regardless of the Saito primer set or sample type, is presented in Table 2 (and Fig 2B). Some patients were negative at early timepoints, but then later became positive, although they did not develop clinical recurrence during the study. This predominated in patients that had received chemoradiation, rather than surgery, but the significance is unclear. In others, positive samples occurred in early time points. This might reflect delayed death and phagocytosis of treated tumor tissue and slow release of HPV DNA into plasma that dissipates over time. The presence of HPV DNA in WBCs suggests a possible phagocytic mechanism. In many cases, the WBC were positive by the matching Saito et al. primer sets as seen in the testing of plasma. This suggests specificity and may support the concept of phagocytosis of tumor tissue. In several cases the WBC sample was positive while the matching plasma was negative. We reason that HPV DNA picked up from dying tumor tissue by circulating phagocytes is more likely to occur in advanced stage patients treated with chemoradiation, rather than in stage I cases treated with surgery alone which presumably removes all tumor tissue. However, comparison of treatments administered with frequency of detection of HPV in the series of blood samples, either plasma or WBC using the Saito et al primer sets, did not bear this out (Table 2). Likewise, it was not correlated with the histology, grade, or stage of cervical cancer (S5 Table). Alternatively, as none of the treatments directly targets the virus, these positives may reflect unresolved HPV16 infections in cervical cancer patients. Since there was no clinical evidence of recurrence by the end of the study, evidence that the HPV DNA detected derives from live circulating tumor cells is lacking.

Notably, there were also multiple sporadic positive samples, including one that corresponded with the sample with a positive result by the cobas HPV test. It is unclear if this represents stochastic release of dead tumor tissue, or intermittent resurgence and immune suppression of de novo disease.

Cobas HPV was also used for testing plasma and a saliva sample obtained from patients with HPV+ HNSCC, and all cases were HPV16+ (Table 3). For the plasma samples, 5 of 17 gave invalid results. HPV16 DNA was detected in 6 plasma samples by the cobas test, including pre-treatment samples of 1 of 2 (50%) stage I cases, 2 of 3 (66%) stage 2 cases, and 3 of 3 (100%) stage 3 cases. During the pre-treatment phase only two samples yielded negative results with cobas HPV; in total 6 of 8 (75%) were positive. The positive cobas tests showed a median Ct value of 35.7 [range: 34.3–37.4]. All five post-treatment patients with no evidence of disease were negative by the cobas test; of these 3 had detectable HPV16 DNA by cobas test in the pre-treatment plasma sample. The single saliva sample was positive by cobas for HPV16, as well as in plasma.

**Table 3:** Samples from HNSCC patients tested with the cobas HPV test and clinical reports of other commercial tests.

Subject #	Plasma	Cobas (Ct)	Pre-/Post-Treatment	Diagnosis	Gender	Race	Ethnicity	Stage
1	Plasma	Not detected	Pre	R tonsil HPV + SCC	M	White	non-hispanic	T2N1 M0
2	Plasma	Not detected	Pre	R tonsil HPV + SCC	F	White	non-hispanic	T1N1 M0, Stage 1
2	Plasma	Not detected	Post	R tonsil HPV + SCC	F	White	non-hispanic	T1N1 M0, Stage 1
3	Saliva	**HPV16 (35.08)**	Pre	BOT HPV + SCC	M	White	non-hispanic	cT3N2b (AJCC 7^th^)
3	Plasma	**HPV16 (34.26)**	Pre	BOT HPV + SCC	M	White	non-hispanic	cT3N2b (AJCC 7^th^)
4	Plasma	**HPV16 (36.10)**	Pre	R. Tonsil Primary HPV + SCC	M	Black	non-hispanic	T2N1M0
4	Plasma	Not detected	Post	R. Tonsil Primary HPV + SCC	M	Black	non-hispanic	T2N1M0
5	Plasma	**HPV16 (37.05)**	Pre	R. BOT/lingual tonsil Primary HPV + SCC	M	White	non-hispanic	T1N1M0
6	Plasma	**HPV16 (35.37)**	Pre	L tonsil HPV + SCC	M	White	non-hispanic	T3N1 M0
6	Plasma	Not detected	Post	L tonsil HPV + SCC	M	White	non-hispanic	T3N1 M0
7	Plasma	Not detected	Post	R tonsil HPV + SCC	M	White	non-hispanic	T1N1 M0
8	Plasma	**HPV 16 (37.41)**	Pre	L glossotonsilar HPV + SCC	M	White	non-hispanic	T2N2b, MX
8	Plasma	Not detected	Post	L glossotonsilar HPV + SCC	M	White	non-hispanic	T2N2b, MX

Cobas data is shown as Ct values. BOT: Base of Tongue; SCC: Squamous cell carcinoma.

## Conclusions

There are currently no FDA-licensed blood-based biomarkers for the early detection of HPV+ cancers or to monitor treatment outcomes. The measurement in plasma of cfDNA-derived from the tumor, and specifically HPV DNA, show great promise for these applications. The most advanced technologies to measure cfHPV DNA are currently dPCR and NGS, and they are generally preferred over qPCR because of their greater sensitivity. While they have great promise [17,18], they have not yet been shown to have clear clinical benefit [19] and are more complex than qPCR.

Since the qPCR-based cobas HPV test is used globally to detect hrHPV DNA in cytologic samples in a high throughput robotic format at relatively modest cost/assay [14], we examined its potential applicability to plasma samples. This assay might be considered to detect or monitor advanced stage HPV+ cervical and oropharyngeal cancers, but we observed several limitations to this application. Firstly, using plasma it did not detect carcinoma in situ or stage I cervical cancers. Secondly, it is not strictly quantitative, although Ct values are available and a standard can be applied. Although we used 1 mL of plasma for testing, sensitivity might be increased if a larger volume of plasma could be processed. Finally, the cobas test targets the L1 gene which might be disrupted during viral integration, rather than E6/E7 that must be retained and expressed in HPV+ cancers. While a positive test would suggest the presence of advanced HPV+ cancer, and therefore provide useful prognostic information, the interpretation of a negative test is much less clear.

Several groups used the Roche cobas 4800 assay to detect hrHPV DNA in oral gargle samples of oropharyngeal cancer patients [20] and women with cervical infections and/or dysplasia [21,22]. Here the Cobas 6800 HPV test detected HPV16 in a saliva sample from an HNSCC patient. It is not clear whether this represents detection of virions or HPV16 + cells, but it suggests potential utility as an approach for screening for persistent oral infections or cancer. Unlike testing for cervical cancer, there is currently no routine viral testing for HPV+ HNSCC in oral samples, such as might be implemented during dental checkups to enhance screening beyond palpating the lymph nodes of the neck for detection of subclinical disease [23]. Part of the challenge is the lack of a known precursor lesion and appropriate treatment to intercept progression, although earlier detection of cancer might improve treatment outcomes [19]. In addition, infection is quite prevalent [24] and may clear on its own [25]. Persistent positives warrant follow-up and potentially measurement of HPV DNA in plasma. However, the development of HPV-specific therapeutics could change this dynamic.

The triple primer sets developed by Saito et al. appear to convey increased sensitivity of HPV16 [15]. However, unlike the cobas HPV test, they were not designed to detect all hrHPV. Furthermore, the use of 3 primer sets increases the opportunity for false positives. Application of the Saito et al. primer sets, but not the cobas HPV test, detected HPV16 DNA in plasma of patients who had received standard of care treatment in the past year or more. Since none of these patients showed clinical evidence of recurrence at study exit, there is no firm evidence that the assays are detecting DNA released from minimal residual disease in plasma or circulating tumor cells in WBC samples. The signal might instead reflect phagocytosis of necrotic tumor tissue killed by chemo/radiotherapy. Since plasma and WBC-associated HPV was also detected in cases treated solely with surgery, the signal may alternatively reflect phagocytosis of pre-cancerous disease at the same or a different site and release of viral DNA into blood. Although this would typically be shed extracorporeally, HPV DNA was detected in WBC of a subset of dysplasia cases using the Saito et al. primer sets (**Table 2**).

Detection of HPV DNA in the plasma samples of untreated cancer patients likely reflects shedding of DNA from tumor apoptosis or necrosis, and phagocytosis. Post-treatment, our findings suggest that testing too soon may result in detection of dead tumor tissue, rather than minimal residual disease, and the possibility of detecting ongoing infections and HPV+ lesions at other body sites should be considered. Shedding of dead tumor appears to continue for many months post-treatment. However, with regular testing it might be distinguished by its downward trajectory in levels as compared to ascending blood HPV DNA levels with recurrence/progression disease. This might be made feasible with a simple, inexpensive qPCR-based assay as compared to more complex sequencing-based approaches. While qPCR testing may be less sensitive than dPCR or NGS-based testing, with regular use and assessment of the trajectory of viral load we speculate that simple qPCR testing might provide clinically useful information.

The cobas HPV testing of plasma detected a higher fraction of oropharyngeal cancer cases as compared to the cervical cancers, and none of the vaginal cancer samples [23]. This may reflect a distinct biology, such as a lower frequency of integration and thus higher viral copy number in the oropharyngeal cancers as well as the site of origin, and a significant fraction of vaginal cancers are not HPV-driven. Nevertheless, in two of 8 pre-treatment samples from early stage HNSCC cases the cobas HPV test was negative. The number of invalid tests in this study highlights the importance of the inclusion of an internal control to detect interference with the qPCR reaction to identify false negatives, sample stability, the potential importance of the phlebotomy process, extraction of plasma, and the process of DNA purification to eliminate PCR inhibitors.

In conclusion, this study provides a proof-of-concept investigation of analytical sensitivity and assay performance in patients with known disease using a non-validated modification of the Roche Cobas 6800 HPV test and the triple primer sets developed by Saito et al. for detection of HPV16. The data address analytical detectability rather than proven clinical utility. Our findings suggest that plasma HPV DNA detection by either assay is unlikely to be sufficiently sensitive for population screening or reliable early detection, particularly given the poor detection rates in stage I disease. Potential applications in treatment monitoring, recurrence surveillance, prognostic stratification, or assessment of minimal residual disease in selected high-risk patients are more promising. However, at this stage, detection of very low levels of HPV DNA does not necessarily imply clinical relevance, particularly in post-treatment patients where intermittent positivity may reflect residual tumor DNA, transient circulating fragments, contamination, or assay noise. The clinical significance of our findings therefore remains uncertain. Limitations of the study include a lack of true comparator gold standard and genomic sequencing data for the pathology samples to provide definitive HPV typing and integration status, small sample sizes, and the relatively limited post-treatment follow-up, with sporadic sampling and no robust longitudinal clinical correlation. Intermittent low-level positivity is difficult to interpret in the absence of recurrence data, imaging correlation, or systematic serial measurements over time.

## Supporting information

S1 Table Detection of HPV16 (A) or HPV18 genomic DNA (B) in plasma with the cobas 6800 HPV test. Data are presented as Ct values. Replicates were tested to determine the analytical sensitivity of the assay.
(DOCX)

S2 Table Detection of HPV+ cell pellets in plasma using the cobas 6800 HPV test. Ct values are shown for positive tests. Replicates were tested to determine the analytical sensitivity of the assay.
(DOCX)

S3 Table Detection of HPV16 DNA in the plasma or WBC of HPV16 + cervical cancer patients. The HPV16 + cervical cancer patients, who had no clinical evidence of disease within 12 months of completed standard of care treatment, were enrolled in a phase I clinical trial testing vaccination with TA-CIN, a fusion protein comprising HPV16 L2E7E6. Blood was collected at baseline (BL) pre-vaccination, and at Months 1, 2, 3 and 6 and Years 1 and 2 (M1, M2, M3, M6, Y1, Y2 respectively). Samples of plasma (1mL) or WBC were tested with either the cobas 6800 test or the three primer sets of Saito et al. (E7, BR E1-5 and HRE5L2-4). §Negative on re-test of another aliquot.
(DOCX)

S4 Table Proportion positive and concordance of detection of HPV16 DNA in the plasma (A) or WBC (B) of HPV16 + cervical cancer patients from a clinical trial (NCT02405221) using the Saito et al primer sets. (DOCX)

S5 Table Association between changes from baseline in the highest intensity of plasma or WBC samples, measured using the Saito et al. primer sets at each follow-up time point, and disease characteristics including site, histology, grade, stage, and prior therapy (chemotherapy and/or radiation vs. surgery only) among HPV16-positive cervical cancer patients enrolled in a clinical trial (NCT02405221). (DOCX)
