# Supplementary material for: Application of Cobas 6800 HPV testing and qPCR utilizing three genomically-optimized primer sets for detecting HPV DNA in the blood of HPV Positive dysplasia and cancer patients
Source: PLoS One. 2026 Aug 25;21(8):e0354671. doi: 10.1371/journal.pone.0354671 (PMC13505944; doi:10.1371/journal.pone.0354671)
Supplement: Supplementary file 1 [file pone.0354671.s001.docx]

**S1 Table. Detection of HPV16 (A) or HPV18 genomic DNA (B) in plasma with the cobas 6800 HPV test.** Data are presented as Ct values. Replicates were tested to determine the analytical sensitivity of the assay.

**A.**

| **Other hrHPV** | **HPV16** | **HPV18** | **Copies HPV16 genome/mL** |
| --- | --- | --- | --- |
| Neg | 32.2 | Neg | 1000 |
| Neg | 32.76 | Neg | 1000 |
| Neg | 32.44 | Neg | 1000 |
| Neg | 35.19 | Neg | 100 |
| Neg | 35.6 | Neg | 100 |
| Neg | 34.99 | Neg | 100 |
| Neg | 36.39 | Neg | 10 |
| Neg | 37.48 | Neg | 10 |
| Neg | Neg | Neg | 10 |
| Neg | 37.08 | Neg | 1 |
| Neg | 38.43 | Neg | 1 |
| Neg | 37.74 | Neg | 1 |
| Neg | Neg | Neg | 0.1 |
| Neg | Neg | Neg | 0.1 |
| Neg | Neg | Neg | 0.1 |
| Neg | Neg | Neg | 0.01 |
| Neg | Neg | Neg | 0.01 |
| Neg | Neg | Neg | 0.01 |

**B.**

| **Other hrHPV** | **HPV16** | **HPV18** | **Copies HPV18 genome /mL** |
| --- | --- | --- | --- |
| Neg | Neg | 33.52 | 1000 |
| Neg | Neg | 33.58 | 1000 |
| Neg | Neg | 33.52 | 1000 |
| Neg | Neg | 35.82 | 100 |
| Neg | Neg | 35.98 | 100 |
| Neg | Neg | 36.6 | 100 |
| Neg | Neg | Neg | 10 |
| Neg | Neg | Neg | 10 |
| Neg | Neg | Neg | 10 |
| Neg | Neg | Neg | 1 |
| Neg | Neg | Neg | 1 |
| Neg | Neg | Neg | 1 |
| Neg | Neg | Neg | 0.1 |
| Neg | Neg | Neg | 0.1 |
| Neg | Neg | Neg | 0.1 |
