# Supplementary material for: Application of Cobas 6800 HPV testing and qPCR utilizing three genomically-optimized primer sets for detecting HPV DNA in the blood of HPV Positive dysplasia and cancer patients
Source: PLoS One. 2026 Aug 25;21(8):e0354671. doi: 10.1371/journal.pone.0354671 (PMC13505944; doi:10.1371/journal.pone.0354671)
Supplement: Supplementary file 2 [file pone.0354671.s002.docx]

**S2 Table. Detection of HPV+ cell pellets in plasma using the cobas 6800 HPV test.** Ct values are shown for positive tests. Replicates were tested to determine the analytical sensitivity of the assay.

| **Copies/ mL** | **Other hrHPV** | **HPV16** | **HPV18** |
| --- | --- | --- | --- |
| 100 | 29.55 | 32.24 | 31.53 |
| 100 | 30.15 | 33.49 | 32.61 |
| 100 | 29.05 | 33.35 | 30.16 |
| 100 | 29.45 | 32.84 | 31.98 |
| 100 | 29.73 | 31.26 | 31.02 |
| 100 | 29.83 | 31.9 | 31.83 |
| 100 | 29.38 | 32.2 | 30.87 |
| 10 | 31.04 | 33.69 | 31.06 |
| 10 | 31.4 | 32.43 | 31.89 |
| 10 | 31.17 | 35.87 | 32.48 |
| 10 | 30.74 | 35.52 | 30.81 |
| 10 | Neg | 37.15 | 32.16 |
| 10 | 33.84 | 33.64 | 35.58 |
| 10 | 29.92 | 35.37 | 34.51 |
| 10 | Neg | 37.11 | 36.21 |
| 10 | 31.98 | 36.15 | 35.14 |
| 10 | 31.59 | 31.39 | 32.69 |
| 10 | 34.01 | 32.86 | 35.08 |
| 1 | Neg | Neg | Neg |
| 1 | Neg | 37.78 | 37.96 |
| 1 | Neg | Neg | 36.78 |
| 1 | Neg | 33.25 | Neg |
| 1 | Neg | Neg | Neg |
| 1 | Neg | 36.96 | 36.88 |
| 1 | Neg | 37.37 | 36.67 |
| 1 | 30.62 | Neg | 35.88 |
| 1 | Neg | Neg | Neg |
| 1 | Neg | 37.79 | 36.67 |
| 1 | Neg | Neg | Neg |
| 1 | Neg | 37.97 | 36.98 |
