# Supplementary material for: Application of Cobas 6800 HPV testing and qPCR utilizing three genomically-optimized primer sets for detecting HPV DNA in the blood of HPV Positive dysplasia and cancer patients
Source: PLoS One. 2026 Aug 25;21(8):e0354671. doi: 10.1371/journal.pone.0354671 (PMC13505944; doi:10.1371/journal.pone.0354671)
Supplement: Supplementary file 3 [file pone.0354671.s003.docx]

**S3 Table. Detection of HPV16 DNA in the plasma or WBC of HPV16+ cervical cancer patients.** The HPV16+ cervical cancer patients, who had no clinical evidence of disease within 12 months of completed standard of care treatment, were enrolled in a phase I clinical trial testing vaccination with TA-CIN, a fusion protein comprising HPV16 L2E7E6. Blood was collected at baseline (BL) pre-vaccination, and at Months 1, 2, 3 and 6 and Years 1 and 2 (M1, M2, M3, M6, Y1, Y2 respectively). Samples of plasma (1mL) or WBC were tested with either the cobas 6800 test or the Triplex primer sets (E7, BR E1-5 and HRE5L2-4). §Negative on re-test of another aliquot.

| **Subject** | **Study timepoint** | **PLASMA** | **PLASMA**  **Triplex** | | | **WBC**  **Triplex** | | |
| --- | --- | --- | --- | --- | --- | --- | --- | --- |
|  |  | **Cobas (Ct)** | **E7 cp/mL** | **BR E1-5 cp/mL** | **HR E5L2-4 cp/mL** | **E7 cp/mL** | **BR E1-5 cp/mL** | **HR E5L2-4 cp/mL** |
| 1 | BL | neg | neg | neg | neg | neg | neg | neg |
| 1 | M1 | neg | neg | neg | neg | neg | neg | neg |
| 1 | M2 | neg | neg | neg | neg | neg | neg | neg |
| 1 | M3 | neg | 83 | neg | 78 | 4.7 | neg | neg |
| 1 | M6 | neg | neg | neg | neg | **164** | neg | neg |
| 1 | Y1 | neg | **209** | neg | neg | neg | neg | neg |
| 1 | Y2 | neg | neg | neg | neg | 48 | neg | neg |
| 2 | M1 | neg | neg | neg | neg | **146** | neg | **119** |
| 2 | M2 | neg | neg | neg | neg | neg | neg | neg |
| 2 | M3 | neg | neg | neg | neg | neg | neg | neg |
| 2 | M6 | neg | neg | neg | neg | neg | neg | neg |
| 2 | Y1 | neg | neg | neg | neg | neg | neg | neg |
| 2 | Y2 | neg | neg | neg | neg | neg | neg | neg |
| 3 | BL | neg | **754** | neg | neg | neg | neg | neg |
| 3 | M1 | neg | 6.1 | neg | neg | **96** | neg | neg |
| 3 | M2 | neg | **45** | neg | neg | neg | neg | neg |
| 3 | M3 | neg | neg | **2573** | neg | neg | neg | neg |
| 3 | M6 | neg | **95** | neg | neg | neg | neg | neg |
| 3 | Y1 | neg | neg | neg | neg | neg | neg | neg |
| 3 | Y2 | neg |  |  |  | neg | neg | neg |
| 4 | BL | neg | 45 | neg | neg | neg | neg | neg |
| 4 | M1 | neg | neg | neg | neg | neg | neg | neg |
| 4 | M2 | neg | **125** | neg | neg | neg | neg | neg |
| 4 | M3 | neg | neg | neg | neg | neg | neg | 32 |
| 4 | M6 | neg | neg | neg | neg | neg | neg | neg |
| 4 | Y1 | **HPV16 (36.8)** § |  |  |  | 21 | neg | neg |
| 4 | Y2 |  |  |  |  |  |  |  |
| 5 | BL | neg | neg | neg | neg | neg | neg | neg |
| 5 | M1 | neg | neg | neg | neg | neg | neg | neg |
| 5 | M2 | neg | 63 | neg | neg | neg | neg | neg |
| 5 | M3 |  |  |  |  |  |  |  |
| 5 | M6 | neg | neg | neg | neg | neg | neg | neg |
| 5 | Y1 | neg | 75 | neg | neg | neg | neg | neg |
| 5 | Y2 | neg | neg | neg | neg | neg | neg | neg |
| 6 | BL | neg | neg | neg | neg | neg | neg | neg |
| 6 | M1 | neg | neg | neg | neg | neg | neg | neg |
| 6 | M2 | neg | neg | neg | neg | neg | neg | neg |
| 6 | M3 | neg | neg | neg | neg | neg | neg | neg |
| 6 | M6 | neg | neg | neg | neg | neg | neg | neg |
| 6 | Y1 | neg | neg | neg | neg | neg | neg | neg |
| 6 | Y2 | neg | neg | neg | neg | neg | neg | neg |
| 7 | BL | neg | neg | neg | neg | neg | neg | neg |
| 7 | M1 |  | neg | neg | neg | neg | neg | neg |
| 7 | M2 | neg | neg | neg | neg | neg | neg | neg |
| 7 | M3 |  | neg | neg | neg | neg | neg | neg |
| 7 | M6 | neg | neg | neg | neg | neg | neg | neg |
| 7 | Y1 |  |  |  |  |  |  |  |
| 7 | Y2 |  |  |  |  |  |  |  |
| 8 | BL | **neg** | neg | neg | neg | neg | neg | neg |
| 8 | M1 | neg | neg | neg | neg | neg | neg | neg |
| 8 | M2 | neg | neg | neg | neg | neg | neg | neg |
| 8 | M3 | neg | 12 | neg | neg | neg | neg | neg |
| 8 | M6 | neg | neg | neg | neg | neg | neg | neg |
| 8 | Y1 | neg | neg | neg | neg | neg | neg | neg |
| 8 | Y2 | neg | neg | neg | neg | neg | neg | neg |
| 9 | BL | neg | 34 | neg | neg | neg | neg | neg |
| 9 | M1 | neg | neg | neg | neg | neg | neg | neg |
| 9 | M2 | neg | neg | neg | neg | neg | neg | neg |
| 9 | M3 | neg | neg | neg | neg | neg | neg | neg |
| 9 | M6 | neg | neg | neg | neg | neg | neg | neg |
| 9 | Y1 |  |  |  |  | neg | neg | neg |
| 9 | Y2 | neg |  |  |  | neg | neg | neg |
| 10 | BL | neg | **342** | neg | neg | neg | neg | neg |
| 10 | M1 | neg | neg | neg | neg | neg | neg | neg |
| 10 | M2 | neg | neg | neg | neg | neg | neg | neg |
| 10 | M3 | neg | neg | neg | neg | neg | neg | neg |
| 10 | M6 |  | neg | neg | neg | neg | neg | neg |
| 10 | Y1 | neg | neg | neg | neg | neg | neg | neg |
| 10 | Y2 | neg | neg | neg | neg | neg | neg | neg |
| 11 | BL | neg | neg | neg | neg | neg | neg | neg |
| 11 | M1 | neg | neg | neg | neg | neg | neg | neg |
| 11 | M2 | neg | neg | neg | neg | neg | neg | neg |
| 11 | M3 | neg | neg | neg | neg | neg | neg | neg |
| 11 | M6 | neg | 43 | neg | neg | neg | neg | neg |
| 11 | Y1 | neg | neg | neg | neg |  |  |  |
| 11 | Y2 | neg |  |  |  |  |  |  |
| 12 | BL | neg | neg | neg | neg | neg | neg | neg |
| 12 | M1 | neg | neg | neg | neg | neg | neg | neg |
| 12 | M2 |  | neg | neg | neg | neg | neg | neg |
| 12 | M3 | neg | neg | **19,854** | neg | neg | neg | neg |
| 12 | M6 |  |  |  |  |  |  |  |
| 12 | Y1 |  |  |  |  |  |  |  |
| 12 | Y2 | neg |  |  |  |  |  |  |
| 13 | BL |  |  |  |  |  |  |  |
| 13 | M1 |  | neg | neg | neg | neg | neg | neg |
| 13 | M2 | neg | neg | neg | neg | neg | neg | neg |
| 13 | M3 | neg | neg | neg | neg | neg | neg | neg |
| 13 | M6 |  |  |  |  |  |  |  |
| 13 | Y1 | neg |  |  |  |  |  |  |
| 13 | Y2 |  |  |  |  |  |  |  |
| 14 | BL | neg | neg | neg | neg | neg | neg | neg |
| 14 | M1 | neg | neg | neg | neg | neg | neg | neg |
| 14 | M2 | neg | neg | neg | neg | neg | neg | neg |
| 14 | M3 | neg | 80 | neg | neg | neg | neg | neg |
| 14 | M6 | neg |  |  |  | neg | neg | neg |
| 14 | Y1 | neg |  |  |  |  |  |  |
| 14 | Y2 |  |  |  |  |  |  |  |
