# Supplementary material for: Application of Cobas 6800 HPV testing and qPCR utilizing three genomically-optimized primer sets for detecting HPV DNA in the blood of HPV Positive dysplasia and cancer patients
Source: PLoS One. 2026 Aug 25;21(8):e0354671. doi: 10.1371/journal.pone.0354671 (PMC13505944; doi:10.1371/journal.pone.0354671)
Supplement: Supplementary file 4 [file pone.0354671.s004.docx]

**S4 Table.** Proportion positive and concordance of detection of HPV16 DNA in the plasma (A) or WBC (B) of HPV16+ cervical cancer patients from a clinical trial (NCT02405221) using the Triplex HPV16 assay.

| **A.** | **Plasma*** | | | | | | | | |
| --- | --- | --- | --- | --- | --- | --- | --- | --- | --- |
| Assay | **Triplex** | | | | | | | | |
| Primer |  | **E7/or BRE1-5/or HR E5L2-4** | | **E7 cp/mL** | | **BR E1-5 cp/mL** | | **HR E5L2-4 cp/mL** | |
| **time point** | Number of Valid Samples | Concordance (Plasma and  WBC) | Proportion Positive | Concordance  (Plasma and  WBC) | Proportion Positive | Concordance (Plasma and WBC) | Proportion Positive | Concordance (Plasma and WBC) | Proportion Positive |
| **BL** | 12 | 0.67 | 0.33 | 0.67 | 0.33 | 1 | 0 | 1 | 0 |
| **M1** | 14 | 0.93 | 0.07 | 0.93 | 0.07 | 1 | 0 | 0.93 | 0 |
| **M2** | 14 | 0.79 | 0.21 | 0.79 | 0.21 | 1 | 0 | 1 | 0 |
| **M3** | 13 | 0.62 | 0.38 | 0.85 | 0.23 | 0.85 | 0.15 | 0.85 | 0.08 |
| **M6** | 11 | 0.73 | 0.18 | 0.73 | 0.18 | 1 | 0 | 1 | 0 |
| **Y1** | 8 | 0.62 | 0.25 | 0.62 | 0.25 | 0.88 | 0 | 0.88 | 0 |
| **Y2** | 6 | 0.83 | 0 | 0.83 | 0 | 1 | 0 | 1 | 0 |

* No significant difference in positivity rates was observed between the paired Plasma and WBC assays across all time points (McNemar’s test, p > 0.05) [HLT inputs: intuitively to say, the disconcordance between Plasma and WBC are all not significant.]

| **B.** | **WBC** | | | | | | | | |
| --- | --- | --- | --- | --- | --- | --- | --- | --- | --- |
| Assay | **Triplex** | | | | | | | | |
| Primer |  | E7/or BRE1-5/or HR E5L2-4 | | E7 cp/mL | | BR E1-5 cp/mL | | HR E5L2-4 cp/mL | |
| **time point** | Number of Valid Samples | Concordance (Plasma and WBC) | Proportion Positive | Concordance (Plasma and WBC) | Proportion Positive | Concordance (Plasma and WBC) | Proportion Positive | Concordance (Plasma and WBC) | Proportion Positive |
| **BL** | 12 | 0.67 | 0 | 0.67 | 0 | 1 | 0 | 1 | 0 |
| **M1** | 14 | 0.93 | 0.14 | 0.93 | 0.14 | 1 | 0 | 0.93 | 0.07 |
| **M2** | 14 | 0.79 | 0 | 0.79 | 0 | 1 | 0 | 1 | 0 |
| **M3** | 13 | 0.62 | 0.15 | 0.85 | 0.08 | 0.85 | 0 | 0.85 | 0.08 |
| **M6** | 12 | 0.73 | 0.08 | 0.73 | 0.08 | 1 | 0 | 1 | 0 |
| **Y1** | 9 | 0.62 | 0.11 | 0.62 | 0.11 | 0.88 | 0 | 0.88 | 0 |
| **Y2** | 8 | 0.83 | 0.12 | 0.83 | 0.12 | 1 | 0 | 1 | 0 |
