# Supplementary material for: Application of Cobas 6800 HPV testing and qPCR utilizing three genomically-optimized primer sets for detecting HPV DNA in the blood of HPV Positive dysplasia and cancer patients
Source: PLoS One. 2026 Aug 25;21(8):e0354671. doi: 10.1371/journal.pone.0354671 (PMC13505944; doi:10.1371/journal.pone.0354671)
Supplement: Supplementary file 5 [file pone.0354671.s005.docx]

**S5 Table.** Association between changes from baseline in the highest intensity of plasma or WBC samples, measured using the Triplex primer sets at each follow-up time point, and disease characteristics including site, histology, grade, stage, and prior therapy (chemotherapy and/or radiation vs. surgery only) among HPV16-positive cervical cancer patients enrolled in a clinical trial (NCT02405221).

|  | **P-value(pv) of testing changes(_chg) in highest copy number from baseline, Wilcoxon test** | | | | | |
| --- | --- | --- | --- | --- | --- | --- |
| **time point** | pv of changes from BL | pv_chg_test_cervix(cervix vs endcervix) | pv_chg_test_histology(AD or AD+SC vs. SCC) | pv_chg_test_grade(mod/poor vs well) | pv_chg_test_stage (2-3 vs 1) | pv_chg_test_surg (chemo or combo vs surg only) |
| **BL** | NA | NA | NA | NA | NA | NA |
| **M1** | **0.04** | 0.31 | 1.00 | 0.67 | 0.78 | 0.69 |
| **M2** | 0.27 | 0.55 | 0.29 | 0.69 | 0.18 | 0.19 |
| **M3** | 1.00 | 0.87 | 0.47 | 0.88 | 1.00 | 0.62 |
| **M6** | 0.45 | 0.59 | 0.45 | 0.23 | 0.64 | 0.35 |
| **Y1** | 0.35 | 0.39 | 0.79 | NA | 1.00 | 0.14 |
| **Y2** | 0.18 | 0.37 | 0.86 | NA | NA | 0.17 |
